# Modulatory Function of Invariant Natural Killer T Cells in Systemic Lupus Erythematosus

**DOI:** 10.1155/2012/478429

**Published:** 2012-06-13

**Authors:** Yi-Ping Chuang, Chih-Hung Wang, Ning-Chi Wang, Deh-Ming Chang, Huey-Kang Sytwu

**Affiliations:** ^1^Department of Microbiology and Immunology, National Defense Medical Center, Taipei 114, Taiwan; ^2^Department of Otolaryngology-Head & Neck Surgery, Tri-Service General Hospital, National Defense Medical Center, Taipei 114, Taiwan; ^3^Division of Infectious Diseases and Tropical Medicine, Department of Internal Medicine, Tri-Service General Hospital, Taipei 114, Taiwan; ^4^Department of Internal Medicine, Tri-Service General Hospital, Taipei 114, Taiwan

## Abstract

Systemic lupus erythematosus (SLE) is a chronic autoimmune inflammatory disease with complex immunological and clinical manifestations. Multiple organ failure in SLE can be caused by immune dysfunction and deposition of autoantibodies. Studies of SLE-susceptible loci and the cellular and humoral immune responses reveal variable aberrations associated with this systemic disease. Invariant natural killer T (iNKT) cells are a unique subset of lymphocytes that control peripheral tolerance. Mounting evidence showing reductions in the proportion and activity of iNKT cells in SLE patients suggests the suppressive role of iNKT cells. Studies using murine lupus models demonstrate that iNKT cells participate in SLE progression by sensing apoptotic cells, regulating immunoglobulin production, and altering the cytokine profile upon activation. However, the dichotomy of iNKT cell actions in murine models implies complicated interactions within the body's milieu. Therefore, application of potential therapy for SLE using glycolipids to regulate iNKT cells should be undertaken cautiously.

## 1. Introduction

Systemic lupus erythematosus (SLE) is a chronic autoimmune inflammatory disease with complex immunological and clinical manifestations. Reduced immune tolerance and abnormal activation of T and B cells lead to autoantibody production mainly against protein-nucleic acid complexes, such as chromatin, and small ribonucleoprotein particles. These autoantibodies complexed with their cognate self-antigens deposit within capillaries of various organs and subsequently mediate systemic disorders. The commonly affected organs include the skin, heart, kidneys, lungs, joints, and central nervous system. This disease usually begins in the 20–45-year age range, although it can occur at nearly any age. SLE is more common in women than in men (>8 : 1). Studies using animal models suggest a role of estrogens in the disease development. The induction of SLE depends on hereditary factors and environmental agents, and inherited genes, infections, ultraviolet light, and some medications are all involved. In general, triggers causing cell death, inefficient clearance of apoptotic cells, and improper exposure of intranuclear antigens to an uncontrolled immune system are potential causes of SLE [1].

Reduced immune tolerance leading to an overt immune response normally precludes various autoimmune disorders. Regulatory T-cells play important roles in mediating peripheral tolerance and immune cell homeostasis. Among them, the natural killer T (NKT) cells are a unique subset of T lymphocytes. NKT cells, which express both NK1.1 and the T cell receptor (TCR) in humans and most murine models, are heterogenous containing both CD1d-restricted and CD1d-nonrestricted populations. CD1d-restricted NKT cells might recognize glycolipids presented by CD1d for development and activation. Type I NKT cells within the CD1d-restricted population express an invariant TCR in the mouse (V*α*14J*α*18) and human (V*α*24J*α*18) combined with a limited but not invariant TCR*β* chain repertoire (preferentially V*β*8.2, V*β*7, or V*β*2 in the mouse and V*β*11 in human) [2]. These cells are thus classified as invariant NKT (iNKT) cells that account for more than 80% of CD1d restricted NKT cells in mice. Type II NKT cells are also CD1d-restricted; however, they express variable TCR*αβ* chain combination and are difficult to identify. The most potent agonist of CD1d-restricted NKT cells, *α*-galactosylceramide (*α*-GalCer), a synthetic glycolipid similar to that from an extract of marine sponges, is used widely to define the number and function of type I NKT cells [3]. In this paper, we use the term “iNKT cells” to describe CD1d-restricted NKT cells; however, methods used to identify these cells are described in the text when relevant to avoid confusion. 

iNKT cells are innate-like lymphocytes. Immediately upon activation through TCR engagement, iNKT cells secrete a wide array of cytokines and chemokines. These cells also exert cytolytic activity through granzyme B and FasL-induced apoptosis. iNKT cells can upregulate CD80, CD86, and CD40 on antigen-presenting cells (APCs) to mediate downstream immune responses. Therefore, iNKT cells are considered effector cells that bridge the innate and adaptive immune response [4]. iNKT cells are associated with various autoimmune diseases, including type I diabetes experimental autoimmune encephalomyelitis, and arthritis [5]. Studies also indicate that the number and function of circulating iNKT cells decrease in SLE patients although the immunophysiological role of iNKT cells in SLE is unclear.

Various murine lupus models have been used to investigate the effects of the aberrant number and function of iNKT cells on disease activity. MRL/*lpr* mice, which have a defective point mutation in Fas, spontaneously develop inflammatory lesions affecting the skin and kidneys with marked lymphoproliferation and autoantibody production. CD1d-deficient MRL/*lpr* mice show exacerbated skin lesions [6]. The other widely used murine model, NZB/W F1 (BWF1) mice show an increase in activated iNKT cells with age; however, CD1d deficiency accelerates the onset and progression of nephritis [7]. A chemical-induced lupus model showed that exposure to hydrocarbon oils, such as pristane, facilitates SLE progression through an unknown mechanism. CD1d deficiency exacerbated lupus nephritis in this model, suggesting a regulatory role of iNKT cells [8]. 

 In this paper, we discuss recent studies using different murine models to identify the possible roles of iNKT cells in SLE.

## 2. Numerical Deficiency of iNKT Cells in Human SLE

Changes in the number of iNKT cells are associated with many autoimmune disorders in humans, such as SLE, psoriasis, rheumatoid arthritis, and myasthenia gravis. In human SLE, iNKT cell number is measured using various methods.

Measurement of the expression of TCR V*α*24J*α*18 mRNA level indicates that the numbers of invariant TCR V*α*24J*α*18^+^ CD4 CD8 double negative (DN) T cells are reduced in peripheral blood lymphocytes and in the rheumatoid synovium of patients with SLE [9, 10]. Flow cytometry shows that the number of DN NKT cells expressing TCR V*α*24/V*β*11 is lower in the blood of SLE patients than in healthy controls [11]. Because SLE patients develop progressive lymphopenia, the absolute cell number is affected by the reduction in total lymphocyte number. The proportion of iNKT cells can be calculated to determine the level. The frequency of NKT cells (percentages of CD56^+^CD3^+^ T cells among all lymphocytes) is lower in patients with SLE than in controls [12]. Studies using 6B11 monoclonal antibody, which binds specifically to the conserved CDR3 region of the V*α*24J*α*18 TCR [13, 14], have shown that both the percentage and absolute number of iNKT cells are lower in SLE patients than in healthy controls [15]. Another subpopulation of human V*α*24^+^CD8^+^ iNKT cells express mainly CD161 (NK1.1) and recognize CD1d molecule [16], and the cell number of this population is lower in patients with SLE than in healthy controls [17].

iNKT cell deficiency correlates with Systemic Lupus Erythematosus Disease Activity Index (SLEDAI) [15, 18], suggesting that iNKT cells are involved in the control of disease activity. Although immunosuppressive drugs correlated significantly with log-transformed absolute iNKT cell numbers (*P* = 0.036) in one study [15], the direct effect of medication on iNKT cell numbers was excluded because SLE patients without drug exposure had consistently lower iNKT cell numbers than did healthy controls. Another study found no correlation between drug therapy and the proportion of NKT cells [19]. Thus, the reduction in NKT cells in SLE patients does not appear to be a secondary response to drug therapy.

## 3. Functional Deficiency in iNKT Cells in Human SLE

In addition to the reduction in iNKT cells in human SLE, the poor response of iNKT cells to *α*-GalCer has also been demonstrated in SLE patients [11], whose proliferative response of PBMCs was measured in cells cocultured with *α*-GalCer. The magnitude of the responses varied between subjects, and both good and poor responders were prevalent among both patients and healthy controls. However, the proliferation indices were significantly lower in patients than in healthy controls (median 7.5 versus 28.7, *P* < 0.001) [20]. *α*-GalCer potently activated iNKT cells to produce IFN-*γ* and IL-4. The levels of both mRNA and cytokines in the supernatant of *α*-GalCer-induced PBMCs were lower in SLE patients than in healthy controls.

The lower response of iNKT cells results mainly from their impaired function rather than a defect in the presentation ability of CD1d-bearing cells. In one study, the percentages of CD1d^+^ PBMCs and monocytes were similar in SLE patients and healthy controls, and the expression level of CD1d on PBMCs and monocytes was also indistinguishable between SLE patients and healthy controls [20]. To define further the defective function of iNKT cells, sorted antigen-presenting cells (APCs) from patients or controls were cocultured with patients' iNKT cells. CD3^+^6B11^+^ iNKT cells from an SLE patient failed to proliferate upon *α*-GalCer activation in the presence of monocytes from a healthy control, but iNKT cells from a healthy control were expanded successfully in the presence of monocytes from a healthy control [20]. Another study confirmed that V*α*24^+^ DN iNKT cells from nonresponders fail to proliferate in the presence of APCs from responders, whereas APCs from nonresponders could expand iNKT cells from responders [11]. Another study observed an increase in apoptosis of iNKT cells from patients after 7 days of incubation with *α*-GalCer [15], suggesting that the poor response of iNKT cells might partly result from the susceptibility to activation signaling-induced cell death.

Although the CD1d expression level on B cells and CD1d^+^ B cells is significantly lower in patients than in controls, in vitro coculture experiments indicate that monocytes, but not B cells, are effective APCs for iNKT cells [15].

These data show that iNKT cells in SLE patients are dysfunctional and suggest that activating this population may have therapeutic potential. 

## 4. Function of iNKT Cells Associated with SLE Disease

Various murine models have shown the importance of iNKT cells in SLE progression and systemic disorders. These models have been analyzed and described in detail [21, 22]. In this paper, we focus on recent studies that clarify the functions of iNKT cells and their associations with SLE.

### 4.1. Detection of Apoptotic Cells and Triggering of the Immune Response

SLE can cause severe multiple organs failure resulting from autoantibodies induction. These autoantibodies target nuclear antigens that are theoretically inaccessible. It is hypothesized that the inefficient clearance of apoptotic cells is the source of the antigen pool and that secondary necrotic bodies fuel the inflammation [23–25]. Several genetic studies have identified SLE-susceptible loci, such as CRP [26], and C1q [27], which is involved in clearance of dead cells, and these data support the concept that impaired apoptotic cell clearance is involved in SLE. Recent data suggest that cleavage of autoantigens by granzyme B during cytotoxic-T-lymphocytes- (CTL)-induced apoptosis is involved in human systemic autoimmune diseases [28]. Because CTL-induced targets are often pathogen-infected cells, the molecular mimicry between microbial antigens and autoantigens is not the only explanation for the initiation of autoimmunity after infection. 

In one study of C57BL/6 mice, injection with irradiated apoptotic cells induced autoantibody production [29]. In this mouse model, deficiency in iNKT cells exacerbated the effects of the disease by increasing the production of autoantibodies and glomerular deposition of IgG immune complex [30]. Injection of apoptotic cells rapidly upregulated the expression of CD69 in splenic iNKT cells; the number of IFN-*γ*-producing iNKT cells decreased and the number of IL-10-producing iNKT cells increased in the injected mice. Syngenic apoptotic cell transfer into CD19^−/−  ^mice induced iNKT cells to limit the activation of wild-type B but not CD1d^−/−  ^B cells that were adoptive-transferred, respectively, into CD19^−/−^ recipient. The production of both IgM and IgG3 anti-DNA antibodies was reduced. These data suggest that autoreactive B cells can be regulated by iNKT cells triggered by apoptotic cells in a CD1d-dependent manner.

Increased levels of lysophosphatidylcholine and other oxidized lipids are exposed on the outer leaflet of apoptotic cells [31]. Immunization with these apoptotic cells induces the production of IgM that recognizes oxidized lipids. NKT cells may survey the lipid derivatives on apoptotic cells presented by APCs and then mediate immune tolerance. It was shown recently that apoptotic cells with phosphatidylserine exposed on the outer membrane leaflet can rapidly activate iNKT cells through recognition by T-cell Ig-like mucin-like-1 (Tim-1) on iNKT cells [32]. However, airway hyperactivity was observed rather than improved outcome in this model.

### 4.2. Modulation of Antibody Production

The fact that SLE progression can be caused by various abnormal stimuli of lymphocyte activation suggests the presence of high immunoglobulin levels in the plasma of SLE patients. However, as expected for a heritable trait, such as SLE, analysis of the blood from relatives of SLE patients with subclinical phenotypes should more precisely reflect the pathogenic mechanism and the relationships with genetic and cellular aberrations.

High plasma IgG levels have been noted in both patients with SLE and their relatives [12, 19]. The levels of total IgG and anti-dsDNA IgG in patients with SLE and their relatives are associated with a low frequency of V*α*24^+^ iNKT cells. This result suggests that iNKT cells play an important role in the regulation of IgG production. 

Although an inverse relationship between iNKT cells and IgG production has been observed in humans, murine models reveal a dichotomy in the regulation of IgG production by iNKT cells. One study showed that CD1d-reactive iNKT cells contribute to the development of lupus in BWF1 mice by promoting autoantibody production by B cells [7]. Another study showed that purified iNKT cells but not conventional T cells augment the in vitro secretion of IgM, IgG, and anti-dsDNA antibodies by BWF1 B cells [33] and that CD1d and CD40 are indispensable for this interaction. In addition, adoptive transfer into irradiated *nu*/*nu* BALB/c mice of T cells from the spleen of transgenic BALB/c mice expressing the TCR V*α*4.4J*α*24 and V*β*9 chain recognizing CD1d on syngenic B cells induced lupus and severe immune complex glomerulonephritis, including the production of anti-dsDNA antibodies, in the host mice [34]. 

Another view suggests that iNKT cells have a suppressive role in the regulation of IgG production. In a model using heterozygous J*α*18^+/−^ mice, which show similar pathophysiology to human SLE by having a reduced rather than complete absence of iNKT cells, the mice had a significantly higher anti-dsDNA IgG level and increased activation of autoreactive B cells [30]. Pristane-injected BALB/c mice showed increased autoantibody production and exacerbated nephritis [35, 36]. Further studies of mice with chemically induced diseases examine that the deficiencies in CD1d-restricted cells contribute to the disease. 

In lipopolysaccharide-activated mouse models, reconstitution of active V*α*14^+^ iNKT cells in J*α*18^−/−^ BALB/c mice downregulated anti-dsDNA antibody and rheumatoid factors production but did not change total IgG levels [37]. iNKT cells increased total IgG production and the appearance of activation markers on B cells through soluble mediators and helper T cells, whereas autoreactive B cells were impaired in a contact- and CD1d-dependent manner. This highlights the ability of iNKT cells to distinguish autoreactive from nonautoreactive B cells. Differences in CD1d expression on autoreactive and nonautoreactive B cells suggest differences in regulation between these cells because CD1d expression is higher on dsDNA-responsive autoreactive B cells.

The potent agonist of iNKT cells, *α*-GalCer, is used widely to study the effect of iNKT cells in various disease models. With the administration of C8-*α*-GalCer (with an 8-carbon acyl chain), which skews the serum cytokine secretion toward a Th2 pattern, 50% of BWF1 mice developed lupus nephritis by 30 weeks. And 50% of control BWF1 mice developed proteinuria by about 36 weeks [38]. In contrast to *α*-GalCer, injection of *β*-galactosylceramide, a 12-carbon acyl chain containing glycolipid which rapidly reduced the ratio of iNKT cells in the liver and spleen [39], ameliorated lupus and reduced anti-dsDNA IgG2a production. This implies a complicated role of iNKT cells during the progression of autoimmunity and that alternative agonists of iNKT cells produce different outcomes in murine SLE models. 

### 4.3. Modulation of the Cytokine Profile

Abnormal cytokine profiles have been implicated in the loss of immune tolerance and in a variety of autoimmune diseases. Type I NKT cells produce variety of proinflammatory cytokines, including Th1-, Th2-, and Th17-related cytokines. However, the pathophysiology of human SLE is contradictory to be related to the cytokine alteration by NKT cells in patients. Although early reports demonstrated defective Th1 and excessive Th2 responses in lupus [40], recent data suggest that the levels of both Th1 (IFN-*γ*, IL-12, and IL-18) and Th2 (IL-4, and IL-10) cytokines are increased in the sera of lupus patients [41, 42]. Intracellular cytokine staining reveals comparable IL-4- and IFN-*γ*-expressing lymphocytes in PBMCs from SLE patients without nephritis and healthy donors [43, 44]. However, in a subgroup of patients with severe lupus nephritis, the intracellular cytokine ratio shifts to a Th1 phenotype [44, 45]. In disease-alleviated SLE patients, decreased IFN-*γ*-producing cells and increased IL-4-producing CD4^+^ T cells were observed after corticoid treatment [43] and low-dose UV phototherapy [46], respectively. Although Th1/2-related cytokines might contribute to SLE progression and severity, the cytokine profiles of activated iNKT cells from SLE patients are yet to be determined. 

In addition to Th1- and Th2-related cytokines, iNKT cells can also express IL-17 and IL-21 [47, 48]. IL-17 has recently been implicated in the pathogenesis of SLE [49]. Evidence indicates that production of IL-17 is abnormally high in sera of SLE patients [42] and is correlated with SLE disease severity [49, 50]. When activated by IL-17, the PBMC of patients with lupus nephritis produced higher level of total IgG, anti-dsDNA IgG, and IL-6 [51]. IL-17 production is also high in murine models affected by lupus nephritis [52–55]. It shows spontaneously developed germinal centers in the spleen where IL-17^+^ T cells colocalize with IL-17R^+^ B cells [55] providing the suggestion that IL-17^+^ T cells impact B cells in lupus disease. The main source of IL-17 in SLE patients derives from double negative (DN) TCR*αβ* ^+^CD4^−^CD8^−^T cells [56]. DN T cells are scarce in healthy individuals, but they expand in peripheral blood of SLE patients and infiltrate into kidney with lupus nephritis where they produce proinflammatory cytokines, including IL-17, IL-1*β*, and IFN-*γ* [56–58]. Also in lupus murine models, DN T cells are important IL-17 producer [52]. It also demonstrates elevated plasma levels of IL-21 as well as percentages of IL-21 expressing T cells in SLE patients compared with healthy controls [59, 60]; nevertheless, there is no correlation between IL-21 and disease severity or anti-ds DNA titers [59].

The study of CD1-lipid reactive T cells is much more complicated in humans than in mice. In addition to CD1d, CD1a-, b-, and c-restricted T cells in humans are relatively diverse with CD4^+^, CD8^+^, or CD4^−^CD8^−^ double negative (DN) populations. Although V*α*24 DN NKT cells are numerically decreased in SLE patients, the influence of the subsets of other CD1-lipid reactive T cells on SLE pathogenesis in humans should be further investigated.

In murine models, treatment of adult BWF1 mice (age 8–12 weeks) with *α*-GalCer exacerbated the disease activity, whereas treatment of young BWF1 mice (age 4 weeks) ameliorated SLE symptoms [61]. Moreover, transfer of NK1.1^+^ T cells from aged SLE mice to young BWF1 mice (before the onset of renal failure) induced proteinuria and swelling of the glomeruli. It has been indicated that iNKT cells expand in aged BWF1 mice and the authors reported that *α*-GalCer administration induced predominant IFN-*γ* production in old mice [7]. Use of a blocking anti-CD1d monoclonal antibody to treat BWF1 mice augmented the Th2 responses and ameliorated lupus [61]. These results suggest that the impact of *α*-GalCer treatment on disease in BWF1 mice varies with age and imply that the cytokine profile of iNKT cells influences the progression of SLE. 

In pristane-induced nephritis models, the effect of *α*-GalCer differs between mouse strains. In BALB/c mice, Th2 responses are induced by treatment with *α*-GalCer, which protects mice against nephritis. Conversely, in SJL/J mice, treatment with *α*-GalCer increases the Th1 responses and exacerbates disease [62]. The differences in the effect of *α*-GalCer seem to correlate with the cytokine profile produced by activated iNKT cells. It is the common regulatory mechanism in several autoimmune diseases, such as experimental autoimmune encephalomyelitis, and type 1 diabetes. 

iNKT cells mediate various immune responses, including maintenance of self-tolerance, tumor surveillance, and the response to microbial pathogens. Given the limited TCR diversity, attention has focused on the mechanisms underlying the activation of iNKT cells [63]. In addition to the microbial glycolipid antigens engaging directly with the invariant TCR on iNKT cells [3], indirect activation of iNKT cells by cytokines or endogenous antigen presentation through microbial-stimulated dendritic cells (DCs) is also possible [64–66]. This may explain the ability of various stimuli to activate iNKT cells in the body and implies that iNKT cells might mediate both beneficial and detrimental outcomes depending on the milieu produced by the activated DCs.

The beneficial roles of iNKT cells are involved in immune tolerance and can ameliorate or prevent tissue inflammation [67, 68]. The suppressive effect is mediated globally through tolerogenic DCs, B cells, or regulatory T cells or directly by skewed cytokine production and induction of apoptosis through Fas-FasL engagement of autoreactive lymphocytes [69]. SLE patients have reduced proportions and functions of iNKT cells, which imply that the suppressive effect is mediated by this population. However, a reduced population of iNKT cells cannot be a diagnostic clinical marker of SLE because the frequency of iNKT cells varies markedly between healthy people. Although the suppressive effect was identified recently in a murine lupus model, the function of iNKT cells in humans needs to be clarified. 

Long-term anergy of iNKT cells by reactivation can be induced in mice [70]. The unresponsiveness to *α*GalCer includes reduced proliferative activity and failure of IFN-*γ* production. This suggests that the aberrant proportion and function of iNKT cells in SLE patients may reflect only the outcome after repeated exposure to cognate self-antigens. By contrast, Green et al. did not exclude the possibility that the reduced level of iNKT cells results from attack by upregulated antibody in SLE patients [12]. Therefore, iNKT cells may be a potential therapeutic target in the treatment of SLE patients, although the complicated interactions between iNKT cells and other immune cells and the exact function of iNKT cells require further consideration. 

## 5. Conclusion 

**Figure 1:**
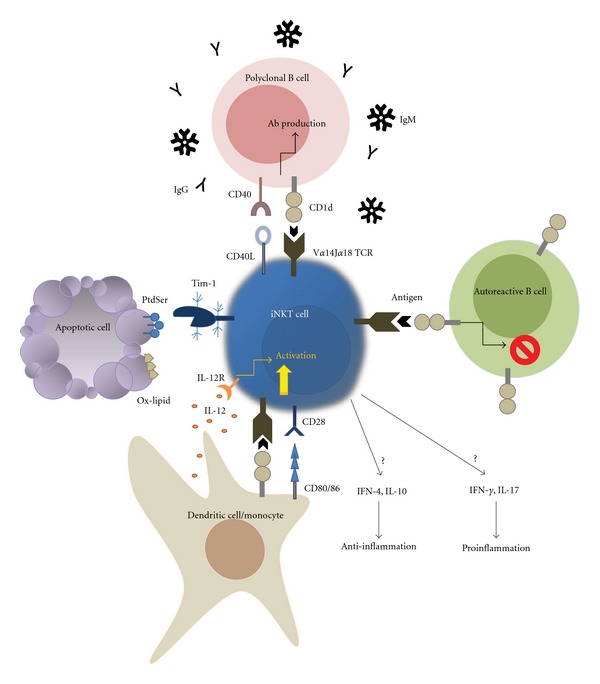
The function of iNKT cells in murine lupus models. iNKT cells in the mouse that express invariant TCR, V*α*14J*α*18, are CD1d-restricted T lymphocytes. The antigens presented by CD1d can be microbial components, endogenous antigen, iGb3, or oxidized lipid (Ox-lipid) derivatives from apoptotic cells. DCs and monocytes are potent APCs that activate iNKT cells both directly through TCR engagement and indirectly through IL-12. Immediately upon activation, iNKT cells release Th1-, Th2-, and T17-related cytokines, depending on the antigen presented and/or the characteristics of the APCs. The proinflammatory cytokines, IFN-*γ* and IL-17, lead predominantly to SLE exacerbation. iNKT cells can sense apoptotic blebs through Tim-1, which recognizes phosphatidylserine (PtdSer) exposed on the outer leaflet membrane, and can mediate immune suppression (see text). By contrast, iNKT cells activate B cells and thus upregulate total IgG and IgM levels in a CD1d-dependent manner, but iNKT cells can also inhibit the activation of autoreactive B cells. CD1d expression levels suggest that iNKT cells are capable of discriminating self- from nonself-reactive B cells.

In this paper, we have discussed the association between iNKT cells and SLE in clinical and murine models. In human SLE patients, the reduced proportion and function of iNKT cells correlate with disease activity and iNKT cells correlate inversely with IgG levels. Recent studies indicate that iNKT cells can sense apoptotic cells and mediate immune tolerance and suggest that iNKT cells can distinguish autoreactive B cells from nonautoreactive B cells to suppress autoreactive antibody production in a CD1d-dependent manner. However, other studies have reported that iNKT cells upregulate total IgG and IgM levels (Figure 1). These findings suggest that iNKT cells are involved in suppressive regulation in SLE.
